# Engineering high-Q superconducting tantalum microwave coplanar waveguide resonators for compact coherent quantum circuit

**DOI:** 10.1038/s41598-025-11744-x

**Published:** 2025-07-25

**Authors:** Shima Poorgholam-Khanjari, Valentino Seferai, Paniz Foshat, Calum Rose, Hua Feng, Robert H. Hadfield, Martin Weides, Kaveh Delfanazari

**Affiliations:** Electronics and Nanoscale Engineering Division, James Watt School of Engineering, University of Glasgow, Glasgow, UK

**Keywords:** Engineering, Materials science, Nanoscience and technology, Optics and photonics, Physics

## Abstract

Tantalum (Ta) has recently received considerable attention in manufacturing robust superconducting quantum circuits. Ta offers low microwave loss, high kinetic inductance compared to aluminium (Al) and niobium (Nb), and good compatibility with complementary metal-oxide-semiconductor (CMOS) technology, which is essential for quantum computing applications. Here we demonstrate the fabrication engineering of thickness-dependent high-quality-factor (high-$\:{Q}_{i}$) Ta superconducting microwave coplanar waveguide resonators. All films are deposited on high-resistivity silicon substrates at room temperature without additional substrate heating. Before Ta deposition, a niobium (Nb) seed layer is used to promote a body-centred cubic lattice ($\:\alpha\:$-Ta) formation. We further engineer the kinetic inductance ($\:{L}_{K}$) of the resonators by varying Ta film thicknesses. High $\:{L}_{K}$ is a key advantage for applications because it facilitates the realisation of high-impedance, compact quantum circuits with enhanced coupling to qubits. The maximum internal quality factor $\:{Q}_{i}$ of $\:\sim\:$3.6 × 10^6^ in the high power regime and $\:{Q}_{i}$ of $\:\sim\:$4.5 × 10^5^ in the single-photon regime is achieved for 100 nm Ta which represents an improvement over previous room-temperature deposited Ta resonators on silicon substrates in the single photon regime, while the highest kinetic inductance of 0.6 pH/sq is obtained for the thinnest film, which is 40 nm. This combination of high $\:{Q}_{i}$ and high $\:{L}_{K}$ highlights the potential of Ta microwave circuits for high-fidelity operation of compact quantum circuits.

**Supplementary Information:**

The online version contains supplementary material available at 10.1038/s41598-025-11744-x.

## Introduction

Superconducting microwave coplanar waveguide (CPW) resonators are one of the fundamental components of circuit quantum electrodynamics (cQEDs), and quantum computing chips owing to their high-quality factor resonances and the simplicity of the fabrication process. Additionally, their characteristic impedance can be tuned by modifying the gaps^'^ width and the centre conductor’s width^1–4^. However, two-level systems (TLSs) and quasi-particle losses significantly affect the performance of superconducting circuits. TLS losses are dominant at low power and temperatures and exist in metal-substrate, metal-air and substrate-air^5–9^ interfaces. Recent efforts have been focused on reducing losses to reach higher quality factors^10–16^. The fabrication of high-performance qubits is highly feasible with superconducting films that exhibit low dielectric losses at surfaces and interfaces^17–20^. Enhancing fabrication quality and minimizing interfaces help to decrease the losses^21–23^. High-$Q$ superconducting resonators, which exhibit both improved coherence and enhanced transmission, are useful for hybrid^24,25^ and unconventional^26^ circuits in emerging quantum applications^27–29^. Tantalum (Ta) is one of the materials that is often used in superconducting circuits and has recently attracted significant attention in the manufacturing of robust superconducting quantum circuits. It offers superior performance due to its low intrinsic losses, relatively high superconducting transition temperature, chemical stability and compatibility with semiconductor manufacturing processes, such as CMOS technology^30–34^. The native oxide layer of Ta is thinner than other materials such as aluminium^35^ and titanium^36^, resulting in lower loss and higher coherence time in superconducting qubits. While aluminium^37^ and niobium^38^ have been extensively investigated for high-Q superconducting resonators, tantalum offers several specific advantages, such as higher oxidation resistance, outstanding chemical stability^39^ in comparison to aluminium, reduced flux-trapping losses and a more robust surface oxide compared to niobium^40^.

The potential of body-centred cubic (BCC) lattice ($\alpha$-Ta)^41^ film as a superconductor for the development of high-performance, large-scale superconducting quantum circuits could facilitate the realization of practical superconducting quantum computers^21^. It can be grown on heated sapphire substrates without a buffer layer^30,42,43^, on heated sapphire substrates with a buffer layer^44^, or on unheated/heated silicon substrates with/without a seed layer^31–33,45,46^. However, sapphire is an insulator and an extremely hard material, making it incompatible with large-scale, conventional CMOS fabrication technologies. The use of advanced integration techniques, such as through silicon vias (TSVs) technology to scale up sapphire substrates for growing $\alpha$-Ta films is challenging. In contrast, silicon substrates are primarily used for large-scale integrated circuits^21,45^.

On the other hand, reducing the thickness of superconducting films increases their kinetic inductance (the inductance arising from the kinetic energy of Cooper pairs), leading to a shift in the resonance frequencies of superconducting resonators. The field of cQED is increasingly influenced by materials with high kinetic inductance. The unique properties of high kinetic inductance materials can open up new possibilities for quantum information processing and applications in sensing and metrology. Moreover, materials with high kinetic inductance are valuable for enhancing device performance in superconducting electronics, quantum computing, and other cutting-edge technologies. These include microwave detectors^47,48^, parametric amplifiers^49,50^, fluxonium qubits^51,52^, resonators^2^, and high-coherence quantum processors^53,54^. The high ${L}_{K}$ attained in thinner films, like 40 nm Ta, directly enables circuit components to have smaller physical dimensions without sacrificing functionality. In qubit designs that are limited by their footprint, this compactness is especially advantageous. In such cases, it is crucial to reduce the size of resonators and interconnects in order to increase qubit density, mitigate crosstalk, and enhance scalability. However, thin films serve additional purposes. For example, in superconducting nanowire single-photon detectors (SNSPDs), thin films are used to suppress the critical temperature, which, in turn, suppresses the superconducting energy gap ($\Delta$). Suppressing the superconducting gap is beneficial for improving photon detection efficiency^55^. High kinetic inductance materials can manipulate the signals with better control and precision in the circuits.

In this work, we use a silicon chip as the substrate and α-Ta as the superconducting material to design and fabricate microwave CPW resonators. Before Ta deposition, we sputtered an Nb seed layer. This process was carried out without heating the substrate, making the approach versatile for a wide range of applications in classical and quantum technologies. We explore the thickness-dependent properties of Ta films with thicknesses of 40 nm, 80 nm, and 100 nm with a particular emphasis on the thinnest sample (40 nm), and demonstrate that thin Ta CPW resonators exhibit a competitive kinetic inductance value compared to previously reported works^30–33,45,46^. Following the fabrication, we analyze the effects of temperature and microwave power on the resonators’ internal quality factors.

## Exprimental results and discussion

Our design comprises three quarter-wavelength CPW Ta microwave resonators, each featuring a central line width of 4 μm and gaps of 2 μm wide. All resonators are coupled to a common transmission line. Before deposition, a (100) oriented silicon wafer with a resistivity of 20 kΩ $\cdot$ cm and a thickness of 525 μm was cleaned with acetone, isopropanol (IPA), and reverse osmosis (RO) water, respectively, to remove any particles and residues. Then, an MP 600 S Plassys sputter system was used to deposit Ta films. Before Ta deposition, a 5 nm Nb seed layer was sputtered to promote the growth of the Ta $\alpha$-phase. MP 600 S Plassys sputter system is a confocal sputtering system based on DC magnetron sputtering principles. The deposition angle was 90°, and the distance between the target and substrate was ~ 100 mm. The base pressure achieved in the Plassys MP600S main chamber prior to tantalum sputtering was 10^−9^ Torr. This ultra-high vacuum base pressure was established before introducing the argon sputtering gas to ensure minimal contamination and optimal phase purity of the deposited α-phase tantalum films. A small amount of impurities (particularly oxygen and nitrogen) can affect the quality of the film and form the $\beta$-phase tantalum. Moreover, our DC measurements (will be discussed in the next sections) confirmed $\alpha$-phase deposition. The ${T}_{c}$ was around 4.1 K, which is characteristic of high-quality α-phase tantalum. We did a pre-sputter process for both Ta (P = 318 W, I = 0.801 A and V = 400 v) and Nb films (P = 228 W, I = 0.8 A and V = 286 v). Then we did 210 s sputtering for Ta with (P = 291 W, I = 0.802 A and V = 360 v) and 45 s sputtering for Nb (P = 195 W, I = 0.8 A and V = 244.7 v) at pressure 3.5 mTorr.

**Fig. 1:**
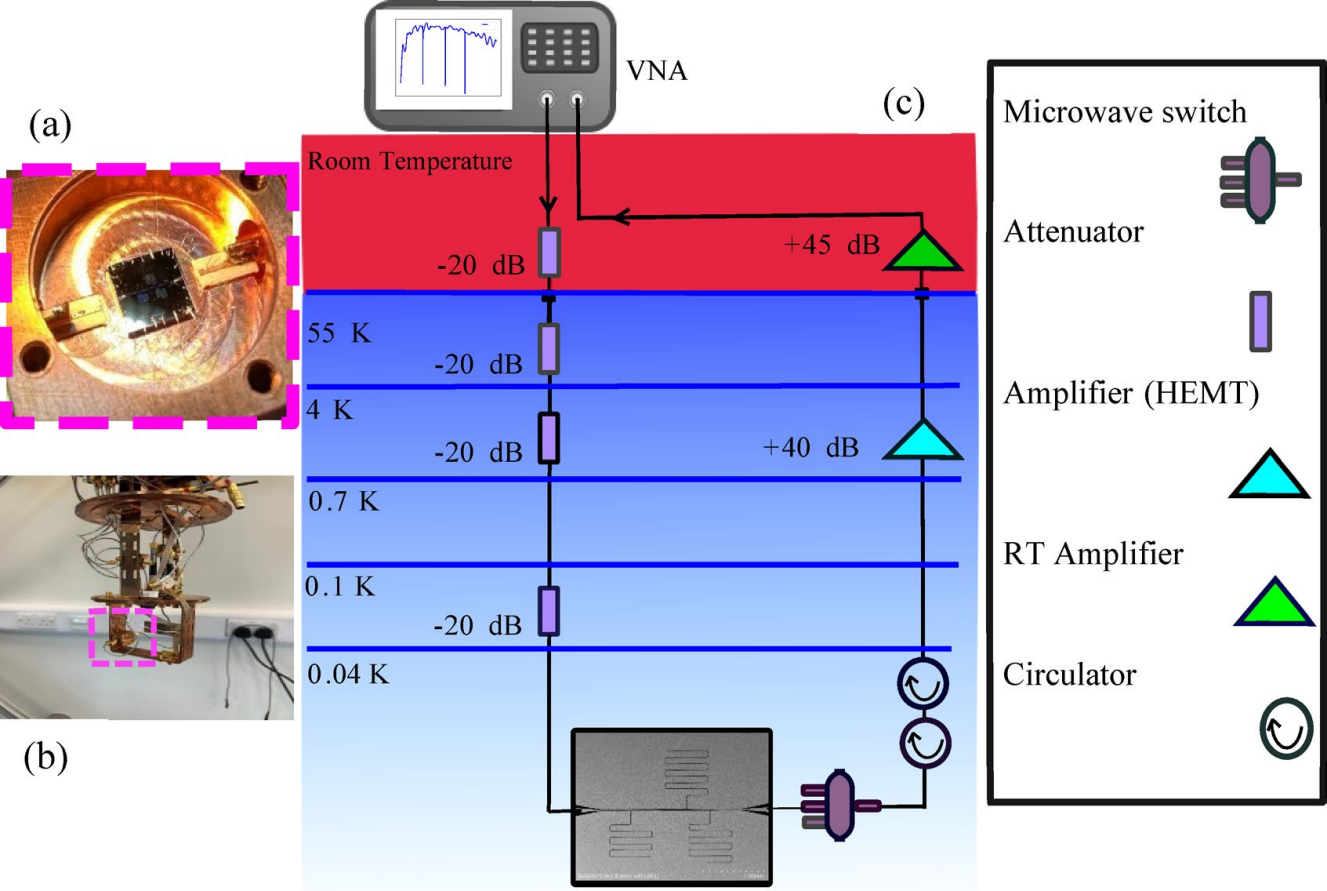
(**a**) The microwave superconducting coplanar waveguide resonators based on 40 nm thick Ta on silicon chip, wire bonded to a copper sample box. (**b**) The packaged Ta microwave superconducting chip is mounted in the lowest stage of a dilution refrigerator. (**c**) Schematic of the cryogenic setup for sub-Kelvin microwave spectroscopy of the chip.

Next, the ZEP520A resist^2^ was spun on the chip and soft-baked at 180 °C for four minutes. Patterns were then formed using electron-beam (e-beam) lithography, followed by dry etching with an ICP 180 etching tool with CF_4_/Ar recipe (CF_4_ = 10 sccm, Ar = 5 sccm with RF power = 10 W and ICP power = 200 W). The resonance frequencies of the resonators were designed to be between 4–8 GHz. Finally, the structures were diced into 5 × 5 mm^2^ chips. One selected chip was wire-bonded to a copper sample box (Fig. 1a) and mounted to an Oxford Instruments Triton 200 Dilution Refrigerator (DR) system (Fig. 1b) where it was cooled down to a base temperature of $T=$ 77 mK. Figure 1c shows the schematic of the DR used for the measurement of the chip. The input signals from the Vector Network Analyzer (VNA) are attenuated by 20 dB at room temperature and 60 dB attenuations inside the fridge before reaching the transmission line of the superconducting circuit. Then after passing through the chip, the output signals were amplified by a high-electron mobility transistor (HEMT) low-noise amplifier with a 40 dB gain at the 4 K stage and by a room temperature (RT) amplifier with a 45 dB gain.

Several methods can be used to extract the resonator parameters such as the internal quality factor (*Q*_*i*_), loaded quality factor (${Q}_{l}$), coupling quality factor (${Q}_{c}$), and resonance frequency (${f}_{r}$) from the measurement data obtained using a VNA. The conventional methods are based on either the amplitude or phase of the ${S}_{21}$^56,57^, while recent methods utilize the full complex scattering data to achieve a more precise calculation of the resonator’s parameters^58–60^. In this work, we employ a notch-type model^59^ to extract the resonator parameters:

1 $$S_{21}^{notch} \left( f \right) = \underbrace {{ae^{i\alpha } e^{ - 2\pi if\tau } }}_{A} \times \underbrace {{ \left( {1 - \frac{{\left( {\frac{{Q_{l} }}{{|Q_{c} |}}} \right)e^{i\varphi } }}{{1 + 2iQ_{l} \left( {\frac{f}{{f_{r} }} - 1} \right)}}} \right)}}_{B}$$

**Fig. 2:**
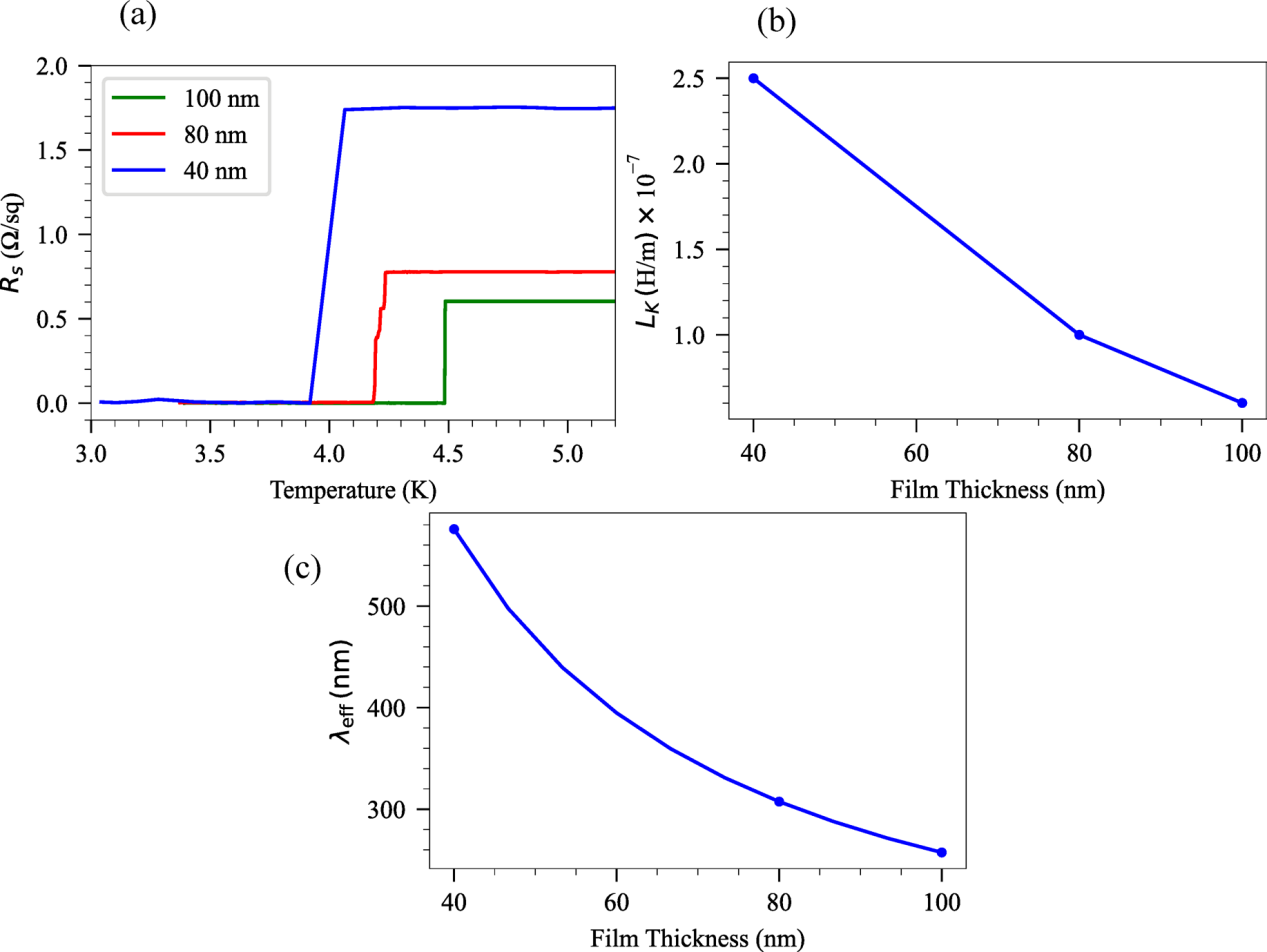
(**a**) Measured sheet resistance ${R}_{s}$ and (**b**) Calculated ${L}_{K}$ (H/m) for Ta with 40 nm, 80 nm and 100 nm thicknesses. (**c**) Calculated penetration depth as a function of film thickness d.

Part *B* of Eq. (1) describes an ideal notch-type resonator, where $f$ represents the probe frequency, *φ* quantifies the impedance mismatch and |${Q}_{c}$| is the absolute value of the coupling quality factor. Part *A* of Eq. (1) defines the environment. Amplitude $a$ shows the cable damping effect in ${S}_{21}$, $\alpha$ a phase shift and $\tau$ represents the electronic delay caused by the length of the cable and the speed of light. We characterized the ${T}_{c}$ of different thicknesses of 40 nm, 80 nm, and 100 nm Ta films (Fig. 2a) by DC measurements. The ${T}_{c}$ of the samples were found to be 4.06 K, 4.2 K and 4.49 K, respectively, confirming high-quality superconducting thin film production. The residual resistance ratios (RRR), summarized in Table 1, indicate good film quality across all thicknesses. Thinner films generally exhibit a higher normal-state resistivity as a result of the increased electron scattering from surfaces, grain boundaries, and structural defects. Thicker films exhibit improved electron–phonon coupling efficiency and higher critical temperatures due to the reduced disorder^61^. Using these critical temperatures and extracting sheet resistances, we could estimate the kinetic inductance (${L}_{K}$) of the Ta samples by using Eq. (2)^2^:

2 $$L_{K} \approx \frac{{\hbar R_{s} }}{{\pi \Delta_{0} }}$$

**Table 1:** Comparison of superconducting microwave CPWs with different thicknesses of Ta films.

Thickness	${f}_{r}$	${Q}_{i}$ at single photon	${Q}_{i}$ at high power	$\frac{1}{{Q}_{TLS}^{0}}$	${Q}_{c}$	${Q}_{l}$	RRR	${v}_{ph}$/${v}_{bulk}$
40 nm	3.654 GHz	2.7 $\times$ 10^5^	1.076 $\times$ 10^6^	6.11 $\times$ 10^–6^	4897	4872	3	0.78
	4.31 GHz	1.6 $\times$ 10^5^	7.3 $\times$ 10^5^	7.3 $\times$ 10^–6^	1045	1043		
	4.88 GHz	2 $\times$ 10^5^	9.1 $\times$ 10^5^	8.4 $\times$ 10^–6^	1455	1453		
80 nm	4.2 GHz	1.1 $\times$ 10^5^	5.5 $\times$ 10^5^	5.4 $\times$ 10^–6^	3459	3438	2.5	0.89
	4.9 GHz	1.5 $\times$ 10^5^	1.82 $\times$ 10^5^	1.4 $\times$ 10^–6^	1415	1405		
	5.64 GHz	5 $\times$ 10^5^	1.85 $\times$ 10^6^	1.5 $\times$ 10^–6^	2664	2660		
100 nm	4.3 GHz	2 $\times$ 10^5^	8 $\times$ 10^5^	5.35 $\times$ 10^–6^	5280	5240	3.5	0.97
	5.1 GHz	1.65 $\times$ 10^5^	2.85 $\times$ 10^5^	2.73 $\times$ 10^–6^	863	860		
	5.8 GHz	4.5 $\times$ 10^5^	3.6 $\times$ 10^6^	1.5 $\times$ 10^–6^	1191	1190		

where $\hbar$, is reduced Planck constant, ${R}_{s}$ is sheet resistance at normal-state and ${\Delta }_{0}$ is the superconducting gap at zero temperature. Assuming ${\Delta }_{0} =$ 1.76 ${k}_{B}{T}_{c}$,^2,62^ where ${k}_{B}$ is the Boltzmann constant, we calculated ${L}_{K}\approx$ 0.6 (pH/sq), 0.25 (pH/sq), and 0.2 (pH/sq) for 40 nm, 80 nm and 100 nm, respectively. Using conformal mapping techniques, by neglecting the ${L}_{K}$, we can estimate ${L}_{l}{=L}_{m}$. As a result, inductance per unit length (${L}_{l}$) and capacitance per unit length $({C}_{l}$ ) of a CPW resonator can be obtained^63,64^, ${L}_{l}=$ 4.13 × 10^–7^ H/m, ${C}_{l}=$ 1.73 × 10^–10^ F/m and ${Z}_{0}=49$ Ω. Then by using ${v}_{ph}=\frac{1}{\sqrt{{C}_{l} \left({L}_{m}+{L}_{k}\right)}}$ and substituting the ${v}_{ph}=\frac{{\omega }_{n}}{{k}_{n}}$ , ${k}_{n}=\frac{\pi }{2l}$ , the ${L}_{K}$ (H/m) can be calculated for all thicknesses (Fig. 2b) shows the calculated ${L}_{K}$ (H/m) for 40 nm, 80 nm and 100 nm Ta film thicknesses. It shows that by decreasing the thickness, the ${L}_{K}$ increases. The reduction in effective phase velocity with decreasing film thickness is given by ${v}_{ph}(t)$/${v}_{ph}^{bulk}=\sqrt{\frac{{L}_{m}}{{L}_{m}+{L}_{k}(d)}}$ where ${v}_{ph}^{bulk}$​ is the phase velocity in a bulk Ta film with negligible kinetic inductance and *d* is the film thickness. The calculated ratios are given in Table 1 and demonstrate that device miniaturization of ~ 20% can be achieved through kinetic inductance engineering in thin superconducting films.

For the Ta film, the effective penetration depth can be obtained by ${\lambda }_{eff}= {\lambda }_{0}$ coth ($\frac{d}{{\lambda }_{0}}$)^44,65^ where ${\lambda }_{0}$ represents the penetration depth of bulk superconductor which is ${\lambda }_{0}=$ 150 nm for Ta^44,66^. Figure 2c shows the calculated penetration depth of fabricated samples. As can be seen, by increasing the thickness, due to a reduction in kinetic inductance, the penetration depth decreases. The fundamental resonance frequency (${f}_{0}$) of the $\frac{\lambda }{4}$ resonators can be obtained using Eq. (3)^42,67,68^ and higher-order harmonics occur at 3 ${f}_{0}$, 5 ${f}_{0}$, 7 ${f}_{0},$ etc.:

3 $$f_{0} = \frac{c}{{4l\sqrt {\varepsilon_{eff} } }}$$

where *c* is the speed of light ($c\approx$ 3 × 10^8^ m/s), $l$ is the length of the resonator, and ${\varepsilon }_{eff}= \frac{{\varepsilon }_{r}+1}{2}$ is the effective permittivity with ${\varepsilon }_{r}$ is the relative permittivity of the substrate.

**Fig. 3:**
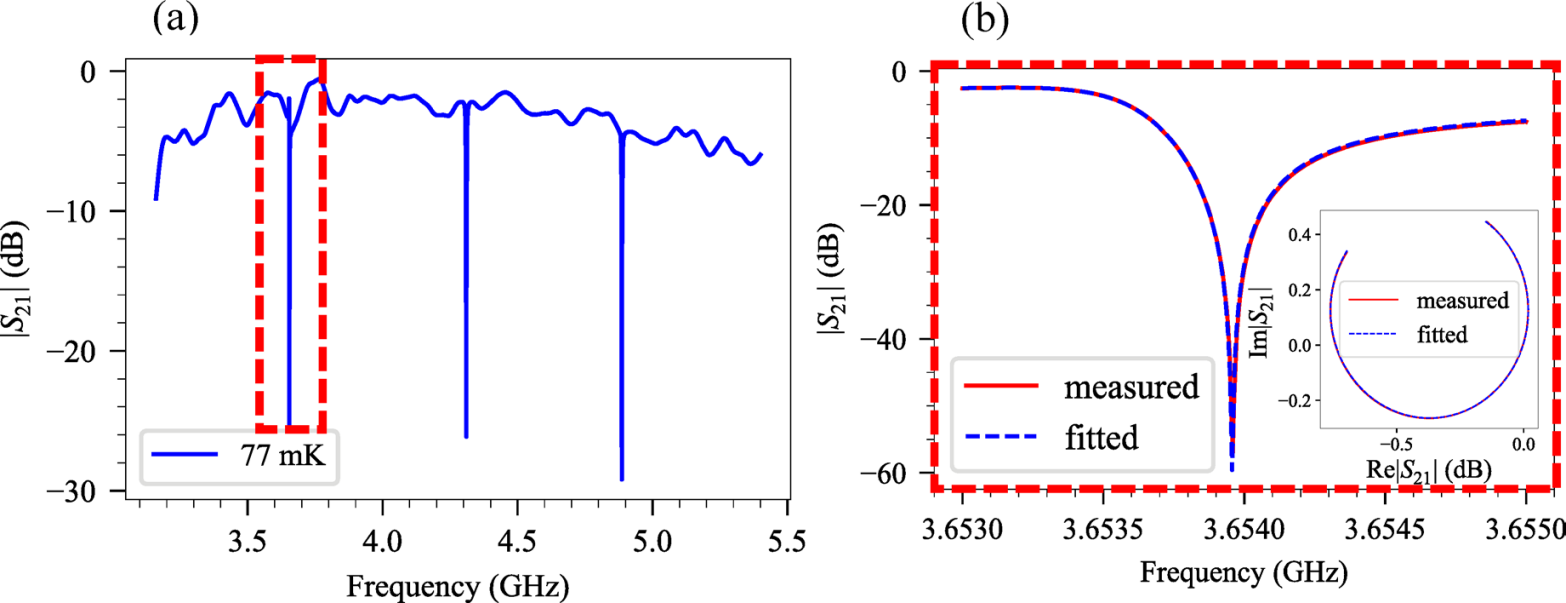
(**a**) Frequency spectrum of the microwave superconducting for 40nm Ta CPW on silicon chip measured at $T=$ 77 mK. (**b**) The magnitude and (inset) circle fit of the measured transmitted signal for ${f}_{r}=$ 3.6539 GHz at high power (VNA power $=$ 0 dBm, corresponding to $<{n}_{ph}> \approx$ 5.52 × 10^5^ with ${Q}_{l}=$ 4872 and ${Q}_{c}=$ 4897 at $T=$ 77 mK.

Figure 3a shows the frequency spectrum of a Ta microwave CPW chip with three resonators, measured at $T=$ 77 mK. The spectrum reveals three resonance frequencies, ${f}_{r}$, within the 3–5 GHz range. The fitted and measured amplitude and circle fit for ${f}_{r} =$ 3.654 GHz at high power (VNA power $=$ 0 dBm) and extracted ${Q}_{l}=$ 4872 and ${Q}_{c}=$ 4897 are shown in Fig. 3b.

**Fig. 4:**
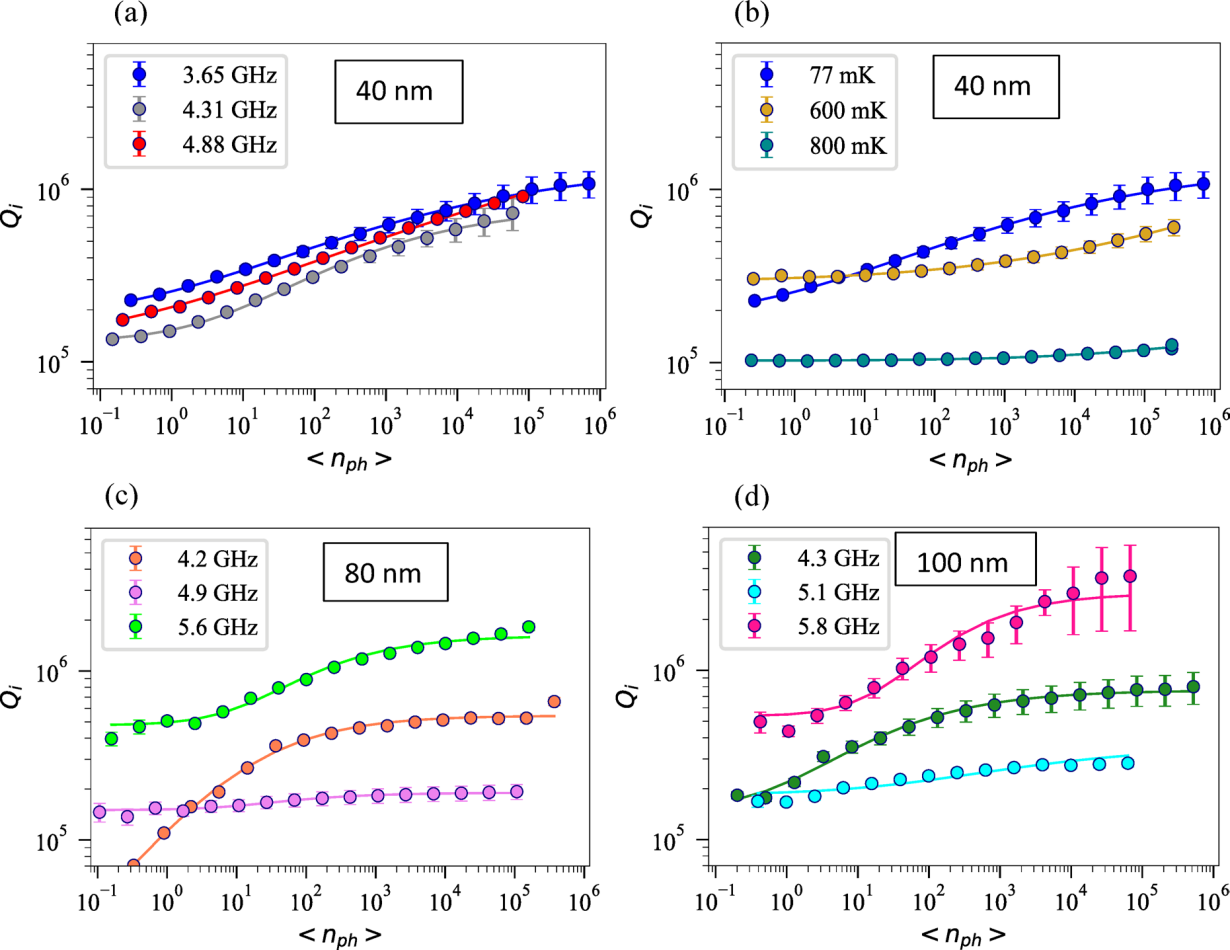
(**a**) Internal quality factor (${Q}_{i}$) of three CPW resonators on silicon as a function of average photon numbers $<{n}_{ph}>$, the scatter plots are for measurement data, solid lines are fitted data based on Eq. (4) and error bars are depicted with caps (**a**) 40 nm Ta at $T=$ 77 mK. (**b**) ${f}_{r}=$ 3.654 at three different temperatures. (**c**) 80 nm Ta at $T=$ 44 mK and (**d**) 100 nm Ta at $T=$ 40 mK.

The photon number inside the $$\frac{\lambda }{4}$$ resonator can be determined by^2,69^:$${P}_{in}={P}_{trans}+{P}_{ref}+{P}_{abs}$$ , $${P}_{ref} =$$ $${P}_{in} \left({\left|{S}_{11}\right|}^{2}\right)$$, $${P}_{trans}={P}_{in} \left({\left|{S}_{21}\right|}^{2}\right)$$, $${P}_{abs}={P}_{in} \left(1-{\left|{S}_{21}\right|}^{2}-{\left|{S}_{11}\right|}^{2}\right)$$ and $$<{n}_{ph}> =\frac{{2Q}_{c}}{{\omega }_{0}} {(\frac{{Q}_{i}}{{Q}_{i}+{Q}_{c}})}^{2}\frac{{P}_{in}}{\hbar {\omega }_{0}}$$ where $${P}_{in}$$ is the input power at the resonator, calculated as ($${P}_{in} = {P}_{VNA}+{P}_{fridge att}+{P}_{RT att} )$$, which $${P}_{fridge att}$$ is the attenuation inside the fridge and $${P}_{RT att}$$ is the room temperature attenuations, $${P}_{ref}$$ is the reflected power, $${P}_{trans}$$ is the transmitted power and $${P}_{abs}$$ is the power absorbed by the resonator. In Fig. 4, the relationship between resonators’ $${Q}_{i}$$ and the average photon number $$<{n}_{ph}>$$ is illustrated. For $${f}_{r}=$$ 3.654 GHz, the $${Q}_{i}$$ is about $$\sim$$ 2.7 $$\times$$ 10^5^ and 1.1 × 10^6^ at single photon and many-photon regimes ($$<{n}_{ph}> =6.93\times {10}^{5})$$, respectively. The variation in $${Q}_{i}$$ is attributed to the TLS loss mechanism. TLS loss is temperature and power-dependent, reaching its maximum at low (milli-kelvin) temperature and low power (single-photon) regime. As power and temperature increase, the TLS loss decreases, which is known as the TLS saturation effect. Thus, an increase in power, leads to a decrease in the TLS loss, resulting the increasing $${Q}_{i}$$. The resonance frequencies $${f}_{r}$$, $${Q}_{i}$$ at single photon and high power regimes, $${Q}_{c}$$, $${Q}_{l}$$ and $$\frac{1}{{Q}_{TLS}^{0}}$$ for all Ta thicknesses of 40 nm, 80 nm and 100 nm are given in Table 1. It can be seen that 100 nm Ta has the highest $${Q}_{i}$$ at high power regimes.

The common TLS model is defined by^70^:

4 $$\delta_{TLS} \left( {T, P} \right) = \frac{1}{{Q_{TLS} }} = \frac{1}{{Q_{TLS}^{0} }}\frac{{\tanh \left( {\frac{{hf_{r} }}{{2k_{B} T}}} \right)}}{{\sqrt {1 + \left( {\frac{{n_{ph} }}{{n_{c} }}} \right)^{\beta } } }}$$

The TLS loss at zero power and temperature $(<{n}_{ph}> =$ 0 and $T=$ 0) is given by $\frac{1}{{Q}_{TLS}^{0}}={{\delta }^{0}}_{TLS}$. Here, ${n}_{c}$ represents the critical photon number, $<{n}_{ph}>$ is the average photon number, and $\beta$ is known to be design-dependent^71,72^.

We fitted the measured data with Eq. (4) for all resonance frequencies and thicknesses at the base temperature $T=$ 77 mK for 40 nm Ta (Fig. 4a), for ${f}_{r}=$ 3.654 GHz at three different temperatures (Fig. 4b), for 80 nm Ta (Fig. 4c) and 100 nm Ta (Fig. 4d). For ${f}_{r}=$ 3.654 GHz (40 nm thickness) at $T=$ 77 mK, we obtained $\frac{1}{{Q}_{TLS}^{0}}=$ 6.11 × 10^–6^ and $\beta =$ 0.44 which indicates the strength of TLS saturation with power^73^ reflecting how effectively the applied power can suppress TLS-induced losses. Our measured *β* values range from 0.4 to 0.9 across different Ta film thicknesses and resonance frequencies. These values are consistent with the literature range for superconducting resonators^74,75^. The frequency dependence observed in Fig. 4c and d suggests that the* β* itself varies with resonance frequency, indicating the distributed nature of TLS energies and their interaction with the electromagnetic field at different frequencies.

The total loss is the sum of TLS loss, quasi-particle loss and ${\delta }_{other}$ (e.g. other losses such as radiation loss, and the finite surface resistance of superconductors loss)^76^:

5 $$\frac{1}{{Q_{i} }} = \delta_{i} = \delta_{TLS} (T, P) + \delta_{qp} (T) + \delta_{other}$$

Figure 4b, presents the comparison of ${Q}_{i}$ as a function of photon number at three different temperatures $T=$ 77 mK and $T=$ 600 mK and $T=$ 800 mK for ${f}_{r}=$ 3.654 GHz, fabricated on 40 nm Ta. It is obvious that the sample at $T=$ 77 mK exhibits the highest ${Q}_{i}$ across the entire power range. As temperature increases, ${Q}_{i}$ decreases significantly, suggesting that the system has reached a regime where quasiparticle losses dominate and TLS saturation effects have stabilized at this reduced level. In accordance with the discussion regarding the transition from TLS losses at low temperatures to quasi-particle losses at higher temperatures, higher temperatures result in lower quality factors as a result of increased quasi-particle density.

The coupling quality factor (${Q}_{c}$) determines the energy exchange between a resonator and its external environment such as measurement setup. Since ${Q}_{c}$ is design-dependent, it is theoretically expected to be constant for a fixed design at all power levels. When ${Q}_{c}$ remains constant and independent of power during the measurement, this consistency demonstrates the stable coupling with minimal influence from the noise or external interference from the measurement setup. In contrast, variable ${Q}_{c}$, indicating that measurement setup or external conditions affect the resonator’s performance, which gives rise to instability or noise^77^.

**Fig. 5:**
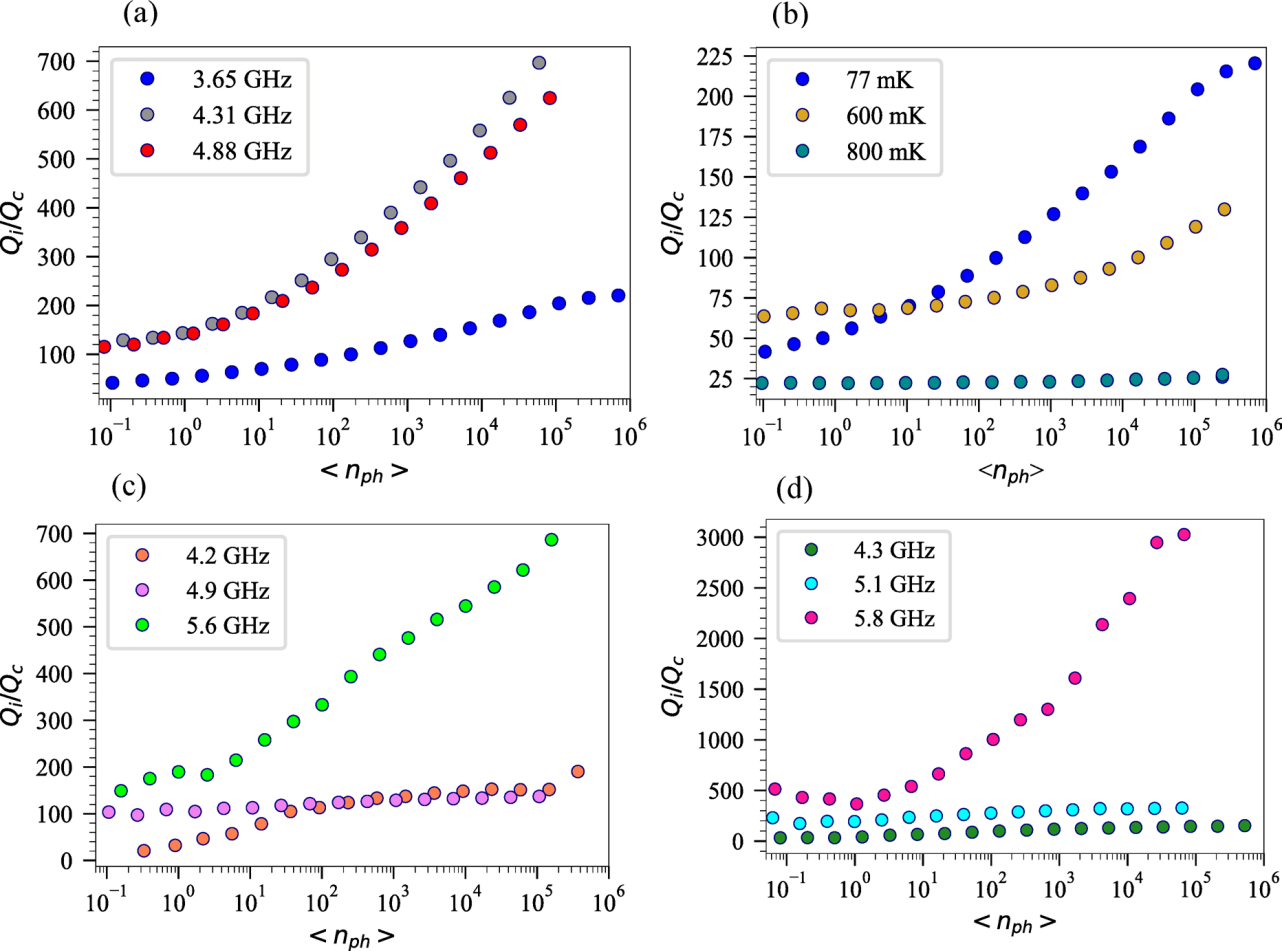
(${Q}_{i}/{Q}_{c}$) of three CPW resonators on silicon as a function of photon number for (**a**) 40 nm Ta, at $T=$ 77 mK. (**b**) 40 nm Ta for ${f}_{r}=$ 3.654 at three different temperatures. (**c**) 80 nm Ta at $T=$ 44 mK, and (**d**) 100 nm Ta at $T=$ 40 mK.

In the overcoupled regime (${Q}_{i}$> > ${Q}_{c}$), the larger fraction of the resonator’s energy couples to the external circuit, making the resonator more susceptible to external noise. However, a constant ${Q}_{c}$, ensures that coupling strength remains stable and measurement results reflect the true resonator behaviour without interference from external variables. Figure 5. shows the ratio of $\frac{{Q}_{i}}{{Q}_{c}}$ for all thicknesses at their resonance frequencies as a function of photon number^6,58,64,78^.

Since the ${Q}_{c}$ is constant, the variation in $\frac{{Q}_{i}}{{Q}_{c}}$ reflect the power-dependent behavior of the internal quality factor.

In this section, the temperature dependence of ${Q}_{i}$ for 40 nm Ta is discussed. As noted in previous sections, the quasi-particle effect is an additional source of temperature-dependent losses. As the temperature increases, the TLS loss becomes negligible, and the quasi-particle becomes the dominant factor. Increasing the temperature results in a rising density of quasi-particles $({n}_{qp}(T))$ leading to a reduction in ${Q}_{i}.$ Consequently, ${n}_{qp}(T)$ determines the losses in the superconducting resonators and the loss model for quasi-particles can be defined by^79^:

6 $$\delta_{qp} (T) = \frac{1}{{Q_{qp} }} = \frac{{\upalpha }}{\pi }\sqrt {\frac{2\Delta }{{hf_{r} }}} \frac{{n_{qp} \left( T \right)}}{{D\left( {E_{F} } \right)\Delta }}$$

where α is the ratio between the kinetic and total inductance of the resonator,$\Delta$ is the superconducting energy gap, ${D(E}_{F}$) is the density of states at the Fermi level and ${n}_{qp}(T$) is the density of a quasi-particle. Additionally, the contribution of quasi-particles can be explained by the Mattis-Bardeen theory^71,80^:

7 $$\delta_{qp} (T) = { }\frac{{2{\upalpha }}}{\pi } = \frac{{e^{ - \varsigma } {\text{sinh}}\left( \xi \right)K_{0} \left( \xi \right)}}{{1 - e^{ - \varsigma } \left( {\sqrt {\frac{2\pi }{\varsigma }} - 2e^{ - \xi } I_{0} \left( \xi \right)} \right)}}$$

where α is the ratio of kinetic inductance to the total inductance of the conductor. ${I}_{0}$ and ${K}_{0}$ are modified Bessel functions of the first and second kind,${\varsigma }=\frac{\Delta }{{k}_{B}T}$ and $\upxi =\frac{{\hbar }\omega }{2{k}_{B}T}$ .

**Fig. 6:**
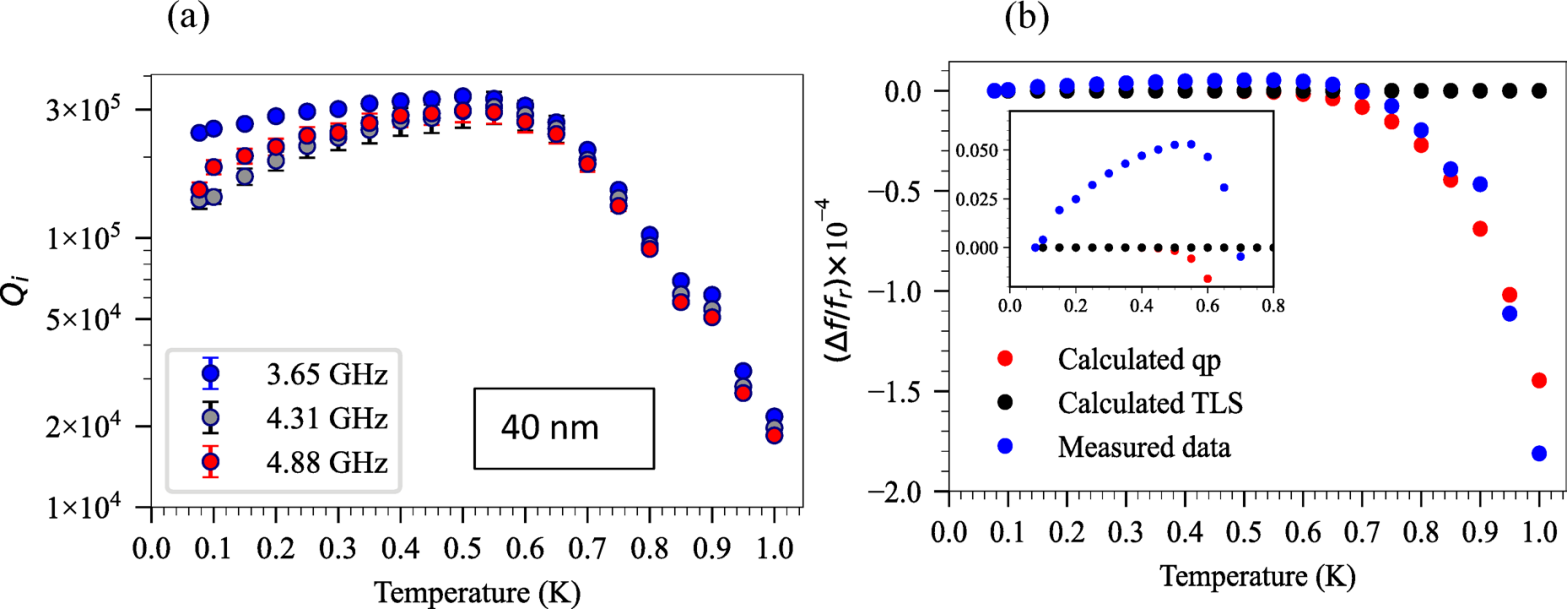
(**a**) Temperature dependence of the ${Q}_{i}$ for three resonators (40 nm Ta) at the single photon regime (error bars depicted with caps at the top and bottom of each data point). (**b**) The measured and calculated shift of resonance frequency as a function of temperature with the zoomed-in view (inset) at the single photon regime for ${f}_{r}=$ 3.654 GHz.

Figure 6a illustrates the dependence of ${Q}_{i}$ on temperature for three resonators in the 40 nm Ta superconducting circuit. As can be seen, at low temperatures, the main factor altering ${Q}_{i}$ is the high TLS loss, resulting in a lower ${Q}_{i}$. As temperature increases, the TLS loss decreases, leading to an increase in ${Q}_{i}$. However, by increasing the temperature to around *T* = 550 mK and beyond, quasi-particle losses become the dominant loss mechanism, causing a subsequent decrease in ${Q}_{i}$. Figure 6(b) shows the measured and calculated resonance frequency shift ($\Delta f$) as a function of temperature. Here, $\Delta f = {f}_{r}\left(T\right)-{f}_{r}(T=$ 77 mK) and $\frac{\Delta f}{{f}_{r}}=\frac{{f}_{r}\left(T\right)-{f}_{r}(T= 77\text{ mK})}{{f}_{r}(T= 77\text{ mK})}$. The red dot shows the calculated data based on Eq. (9), where $\alpha$ is obtained from the ratio of kinetic inductance to the total inductance.

The inset in Fig. 6b represents an increase in resonance frequency of data (blueshift) as the temperature increases from $T=$ 77 mK to $T=$ 550 mK, which is attributed to TLS loss which can be modelled by Eq. (8)^76^. Subsequently, a significant decrease in resonance frequency (redshift) is observed from $T=$ 550 mK to $T=$ 1 K. This redshift results from the increased density of quasi-particles at higher temperatures, leading to an increase in the kinetic inductance and a leftward shift of resonance frequency as described by Eq. (9). Therefore, we can conclude that the total frequency shift is due to both TLS and quasi-particle losses $\Delta f\approx\Delta {f}_{TLS}+\Delta {f}_{qp}.$

8 $${\Delta }f_{TLS} = \frac{1}{{Q_{TLS}^{0} }}f_{r } \frac{1}{\pi } \left( {R\left\{ {{\Psi }\left( {\frac{1}{2} + \frac{{hf_{r} }}{{2\pi ik_{B} T}}} \right)} \right\} - \ln \frac{{hf_{r} }}{{2\pi k_{B} T}}} \right)$$

where Ψ is the digamma function.

9 $${\Delta }f_{qp } = - \frac{1}{2} f_{r } \frac{{{\Delta }L}}{L} = - \frac{1}{2} \alpha f_{r } \frac{{{\Delta }L_{k} }}{{L_{k} }} = - \frac{1}{2} \alpha f_{r } \frac{{\Delta }}{{k_{B} T}}\frac{1}{{\sinh \frac{{\Delta }}{{k_{B} T}}}}$$

The comparison between previously reported superconducting microwave CPW resonators and our work is shown in Table 2. In works reported in references^31–33,46^, Ta films were sputtered on silicon substrates with a seed layer at room temperature. In contrast, in^45^, a heated silicon substrate without a seed layer was used. In works reported in^30,42,43^, Ta was sputtered on the heated sapphire substrate without a buffer layer while in reference^44^, a buffer layer was used.

**Table 2:** Comparison of different approaches for the fabrication of Ta microwave CPW resonators.

References	Substrate	Thickness of SC	Growth Temperature	Metal Preparation	*T* (mK*)*	*Q*_*i*_ single photon	*Q*_*i*_ high power
^33^	Si	150 nm Ta/6 nm Nb seed layer	Room temperature	Sputter	–	–	$\sim$ 10^6^
^45^	Si	100 nm	400–450–500 °C	Sputter	10	0.2–4.5 × 10^6^	55 × 10^6^
^46^	Si	100 nm Ta/5 nm Nb seed layer	Room temperature	–	300	< 10^5^	$\sim$ 10^5^
^30^	Al_2_O_3_	150 nm	550 °C	MBE	10	0.9–1.3 × 10^6^	1.2–2.4 × 10^6^
^31^	Si	200 nm Ta/6 nm Nb seed layer	Room temperature	Sputter	10	–	2 × 10^7^
^42^	Al_2_O_3_	200 nm	750 °C	Sputter	17	10^5^–10^7^	10^7^–2 × 10^8^
^44^	Al_2_O_3_	300 nm/5 nm Nb seed layer	500 °C	Sputter	13	2.5 × 10^6^	27 × 10^6^
^43^	Al_2_O_3_	200 nm	400–500 °C	Sputter	10	0.2–0.67 × 10^6^	0.35–27 × 10^6^
This work	Si	40 nmTa/6 nm Nb seed layer	Room temperature	Sputter	77	1.6–2.7 × 10^5^	0.77–1.1 × 10^6^
	Si	80 nmTa/6 nm Nb seed layer	Room temperature	Sputter	40	1.82–5 × 10^5^	0.55–1.85 × 10^6^
	Si	100 nmTa/6 nm Nb seed layer	Room temperature	Sputter	40	1.65–4.5 × 10^5^	0.8–3.6 × 10^6^

Our study features the thickness-dependent investigation of room-temperature deposited Ta films which shows the ${Q}_{i}$ values of 1.6–5 $\times$ 10^5^ in the single photon regime and places our results within the competitive range of current room-temperature silicon-based approaches while requiring no substrate heating.

## Conclusion

In conclusion, we fabricated and characterized low-loss, high-quality superconducting microwave integrated circuits based on $\alpha$-Ta thin films of various thicknesses, utilizing an Nb seed layer on unheated high-resistivity silicon substrates. Silicon is the preferred choice for a wide range of electronic devices due to its compatibility with advanced silicon wafer technology and CMOS processing, making it highly attractive for scalable quantum devices. The CPW resonators based on $\alpha$-Ta films showed an internal quality factor ${Q}_{i}$ exceeding 8 × 10^5^ at the single photon regime. Additionally, we investigated the effect of temperature and microwave power on ${Q}_{i}$, finding that TLS and quasi-particle losses as the two main factors influencing the resonator properties. Moreover, we characterized the critical temperature and kinetic inductance of Ta films. Our approach is promising for applications in quantum technologies requiring high-*Q*, low loss superconducting circuits compatible with semiconducting circuits.

With thinner films, such as 40 nm Ta, it is possible to reach a high ${L}_{K}$, which directly promotes the reduction of the physical dimensions of circuit components without losing performance. A higher ${L}_{K}$ enables the design of smaller, high-impedance resonators that fulfil the same functions as their larger counterparts. Compactness is particularly advantageous for qubit designs that are constrained by their footprint, as it is crucial to reduce the size of resonators and interconnects. Smaller components not only increase the qubit density, which facilitates greater scalability, but also reduce crosstalk, thereby enhancing the fidelity and stability of quantum operations. This research demonstrates that Ta-based devices are well-suited for next-generation quantum technologies since they allow for small, high-performance quantum circuits.

To further improve the Ta superconducting circuit qualities, future research could focus on improving deposition methods, material purity, and interface engineering, which would increase their usefulness and scalability for realistic CMOS integration.

## Electronic supplementary material

Below is the link to the electronic supplementary material.

Supplementary Material 1.

## Data Availability

Data availability: The datasets analysed during the current study are available from the corresponding author upon reasonable request.
