# Supplementary material for: Engineering high-Q superconducting tantalum microwave coplanar waveguide resonators for compact coherent quantum circuit
Source: Sci Rep. 2025 Jul 25;15:27113. doi: 10.1038/s41598-025-11744-x (PMC12297337; doi:10.1038/s41598-025-11744-x)
Supplement: Supplementary file 1 — Supplementary Material 1. [file 41598_2025_11744_MOESM1_ESM.docx]

**Supplementary**:

1. **DC measurement:**

For critical temperature measurement, we prepared 20×20 mm^2^ bare tantalum film samples for each thickness (40 nm, 80 nm and 100 nm). The samples were selected from the same wafers as the tantalum resonators to maintain identical deposition conditions and film properties. After solvent cleaning of samples, each corner of samples was wirebonded to the copper pads of the sample holder and then mounted to the dilution fridge for vander Pauw measurement. Temperature-dependent resistance measurements were performed during controlled warming from base temperature to room temperature. With this method, the $T_{c}$ of tantalum films was determined which are given before.


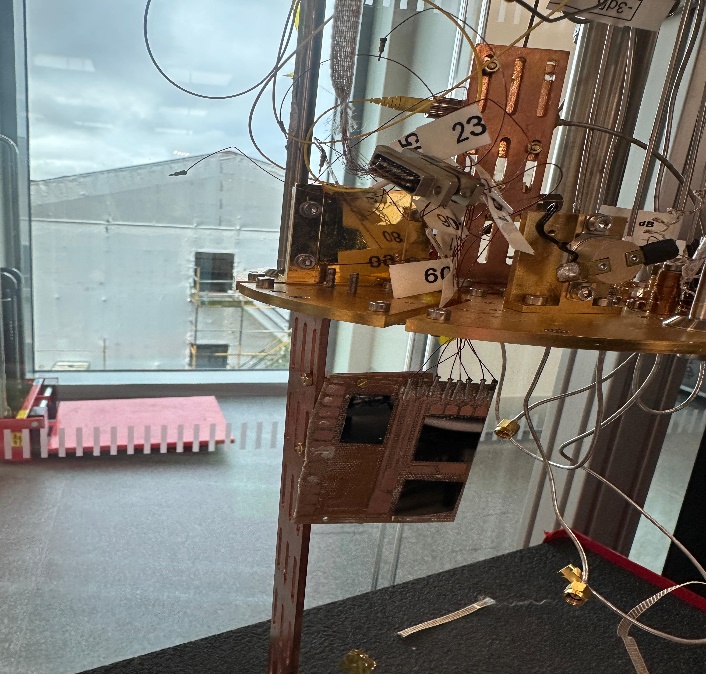


SI Fig.1. Setup of $T_{c}$ measurement

1. Color-plot of amplitude and phase vs temperature

SI Fig. 2 (a) illustrates the colour-coded measured amplitude as a function of frequency in the single photon regime at temperatures ranging from 77 mK to 1K for $f_{r}=$ 3.654 GHz. As the temperature increases from $T=$ 77 mK to $T=$ 1 K, the resonance frequency shifts to lower frequencies and the amplitude diminishes after $T=$ 550 mK. As a result, the measured quality factor decreases. SI Fig. 2 (a) shows the colour-coded representation of phase, mirroring the amplitude's behaviour with a shift towards lower resonance frequencies after $T=$ 550 mK. As temperature increases, due to increase in the density of quasi-particles, the kinetic inductance increases which leads to a shift of resonance frequency to the lower resonance frequencies (redshift).

(b)

(a)


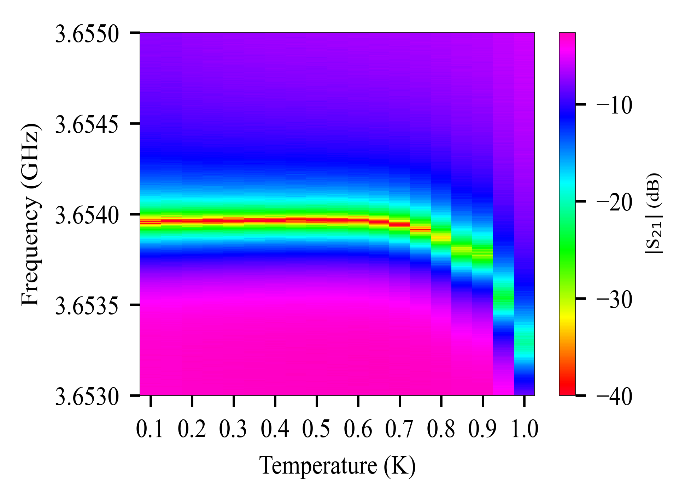

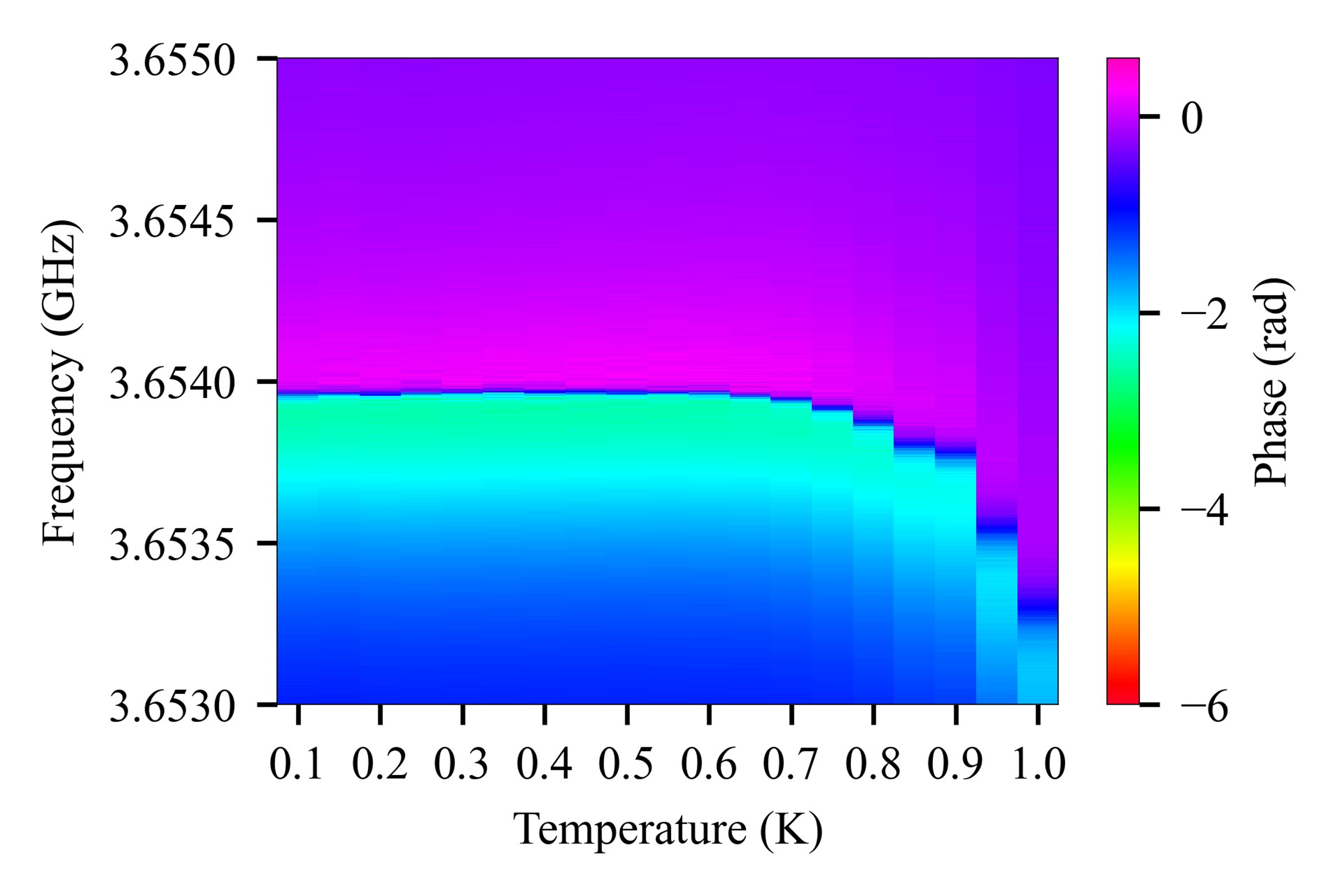


SI Fig 2. Color-map of (a) amplitude (b) phase of $S_{21}$ at different temperatures from $T=$ 77 mK to $T=$ 1 K at single photon regime for 40 nm Ta.
